# Protocol to study the inter-relationship between phageome and lipidome in low-volume preterm milk

**DOI:** 10.1016/j.xpro.2025.103917

**Published:** 2025-06-21

**Authors:** Wen C. Yew, Gregory R. Young, William Cheung, Andrew Nelson, Janet E. Berrington, Darren L. Smith

**Affiliations:** 1 Faculty of Health and Life Sciences, Northumbria University, Newcastle upon Tyne NE1 8ST, UK; 2 Hub for Biotechnology in the Built Environment, Northumbria University, Newcastle upon Tyne NE1 8ST, UK; 3 Faculty of Medical Sciences, Newcastle University, Newcastle upon Tyne NE1 7RU, UK; 4 Newcastle upon Tyne Hospitals National Health Service Foundation Trust, Newcastle upon Tyne NE7 7DN, UK

**Keywords:** Bioinformatics, Microbiology, Molecular Biology, Systems biology

## Abstract

Bacteriophages and lipids in human milk may benefit preterm infant health by modulating gut microbiomes. Here, we present a protocol for analyzing the phageome and lipidome in preterm milk using shotgun metagenomics and untargeted lipidomics approaches, respectively. We describe steps for extracting phages and lipids in low-volume milk, characterizing phageome using an in-house bioinformatic pipeline, and statistical analysis to correlate the phageome and lipidome. Finally, we detail an *in vitro* assay to examine the associations between fatty acid chain length and phage morphotype.

For complete details on the use and execution of this protocol, please refer to Yew et al.^1^

## Highlights

• Phage isolation and DNA extraction from low-volume milk fat and skim milk
• Lipid extraction protocol for LC/MS-based discovery lipidomics
• In-house bioinformatic pipeline to characterize phageome
• *In vitro* assay to study associations of fatty acid chain length and phage morphotype

## Abstract (editor highlights)

Publisher’s note: Undertaking any experimental protocol requires adherence to local institutional guidelines for laboratory safety and ethics.

## Graphical abstract

**Figure:**
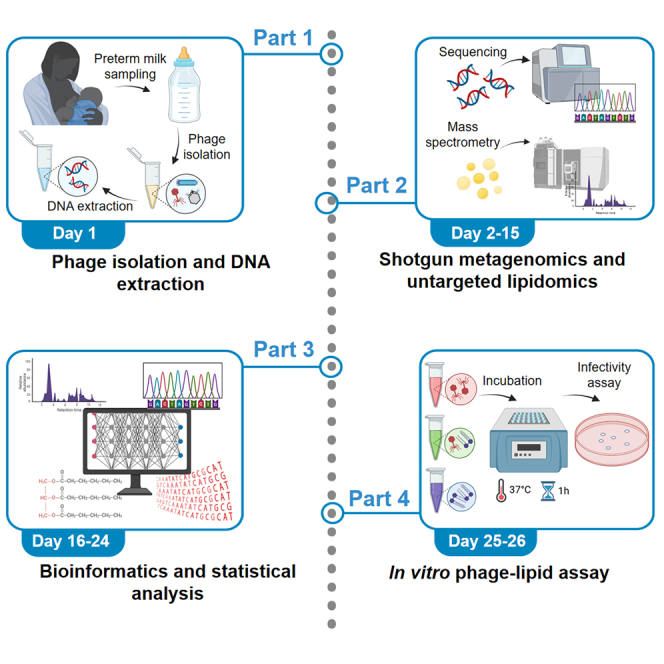

## Before you begin

The protocol below has been optimized to characterize and correlate phageome and lipidome in preterm human milk (HM). The protocol begins from Part 1, which specifically characterizes dsDNA viral communities by omitting steps such as cesium chloride density gradient ultracentrifugation that fails to recover enveloped viruses and those with atypical buoyant densities. We recommend reading Young et al.^2^ to understand the parameters that we tested to establish the viral isolation and DNA extraction method. Sequencing was performed by NUOMICS (Northumbria University, UK) on the Illumina NextSeq (Illumina Inc., USA), using a V2.5 300 cycle chemistry following the manufacturer’s protocol in https://quadram.ac.uk/6-shotgun-metagenomic-illumina-library-preparation/.

Part 2 elaborates the steps in single-phase lipidomic extraction for LC/MS based discovery lipidomics, established by Northumbria Metabolomic Core Service (Northumbria University, UK). All plasticware (tips and tubes) used within the extraction are Eppendorf lo-blind tubes. We recommend using low retention equivalent tips and tubes because lipidomic extraction can and will cause plasticizer leaching. We also encourage the incorporation of extraction blanks as part of the extraction batch for background monitoring and assessment.

Part 3 details the scripts of bioinformatic pipeline established by Northumbria sequencing facility, NU-OMICS to characterize phageome, as well as statistical analysis to correlate phageome and lipidome. We recommend reading Yew et al.^1^ to understand the flow in bioinformatics and statistical analysis before the protocol below.

Part 4 describes the steps of *in vitro* phage-lipid assay to study associations between fatty acid chain length and phage morphotype. We quantified active populations of the *Escherichia coli*-infecting phages Lambda (Sipho-like virus) and T4 (Myo-like virus) by counting plaque-forming units when incubated with their bacterial hosts in the presence of serial dilutions of linoleic acid (a long chain length polyunsaturated fatty acid) running from log units below to log units above physiological concentration.^3^

### Institutional permissions

In this study, bio-banked HM samples were collected from a total of 113 participants (with written consent) who delivered very preterm infants in the Royal Victoria Infirmary Neonatal Intensive Care Unit (NICU). Ethical permission was granted by the National Research Ethics Service (NRES) Committee Northeast – Newcastle and North Tyneside 2 (10/H0908/39). For complete information on the study cohort and HM sampling and storage protocol, please refer to Yew et al.^1^

## Key resources table

**Table:** 

REAGENT or RESOURCE	SOURCE	IDENTIFIER
**Bacterial and virus strains**		

*Escherichia coli*	DSMZ	Catalog: 4230
Phage T4	DSMZ	Catalog: 4505
Phage Lambda	DSMZ	Catalog: 4499

**Biological samples**		

Preterm human milk	RVI NICU	N/A

**Chemicals, peptides, and recombinant proteins**		

Sodium hydroxide solution (0.1 M)	Fisher Chemical	Catalog: 10141860
Hydrochloric acid solution (1 M)	Fisher Chemical	Catalog: 10467640
Phosphate-buffered saline	Sigma-Aldrich	Catalog: 62305
Pepsin from porcine gastric mucosa	Sigma-Aldrich	Catalog: P7000
Lipase from *Rhizopus oryzae*	Sigma-Aldrich	Catalog: 62305
Nuclease-free water	Fisher Scientific	Catalog: 11893933
Norfloxacin	Sigma-Aldrich	Catalog: N9890
TURBO DNase	Invitrogen	Catalog: AM2238
RNase Cocktail Enzyme Mix	Invitrogen	Catalog: AM2286
EDTA solution (0.5 M)	Sigma-Aldrich	Catalog: 3690
Isopropanol	Thermo Scientific	Catalog: 383910025
Proteinase K solution	Invitrogen	Catalog: 25530049
Ammonium formate (10 M)	Fisher Chemical	Catalog: A11550
Calcium chloride (1 M)	Fisher Scientific	Catalog: 10154930
LB broth (Miller)	Sigma-Aldrich	Catalog: L3522
Bacto dehydrated agar	BD	Catalog: 214010
Linoleic acid	Sigma-Aldrich	Catalog: 62230-5ML-F
Optima LC/MS grade water	Fisher Chemical	Catalog: W6500
Dichloromethane (99.9% extra dry stabilize)	Thermo Scientific	Catalog: 326851000
Optima LC/MS grade methanol	Fisher Chemical	Catalog: A4561
Optima LC/MS grade Iso-propanol alcohol	Fisher Chemical	Catalog: A461500
Optima LC/MS grade Acetonitrile	Fisher Chemical	Catalog: A955500
SPLASH Lipidomix Quantitative mass spec internal standard	Merck	Catalog: 330707-1EA

**Critical commercial assays**		

Norgen Phage DNA Isolation Kit	Norgen Biotek Corp.	Catalog: 46850
Beckman Coulter Agencourt AMPure XP beads	Fisher Scientific	Catalog: 10453438
Qubit dsDNA HS Assay Kit	Fisher Scientific	Catalog: Q32851
Nextera XT DNA Library Preparation Kit	Illumina	Catalog: FC-131-1096
NextSeq 500/550 Kit v2.5 (300 cycles)	Illumina	Catalog: 20024908

**Deposited data**		

Raw sequencing data	Yew et al.^1^	ENA: PRJEB58774 https://www.ebi.ac.uk/ena/browser/view/PRJEB58774
Viral reference database	NCBI	https://ftp.ncbi.nlm.nih.gov/refseq/release/viral/ accessed 5 May 2022

**Software and algorithms**		

BBTools	Bushnell^4^	https://jgi.doe.gov/data-and-tools/software-tools/bbtools/
Fastp	Chen et al.^5^	https://github.com/OpenGene/fastp
bioBakery	McIver et al.^6^	http://huttenhower.sph.harvard.edu/biobakery
MEGAN Community Edition	Huson et al.^7^	https://github.com/danielhuson/megan-ce
MEGAHIT	Li et al.^8^	https://github.com/voutcn/megahit
BWA-MEM	Li^9^	https://arxiv.org/abs/1303.3997
SAMtools	Li et al.^10^	http://samtools.sourceforge.net
BEDTools	Quinlan and Hall^11^	http://code.google.com/p/bedtools
VirSorter2	Guo et al.^12^	https://bitbucket.org/MAVERICLab/virsorter2
CheckV	Nayfach et al.^13^	https://bitbucket.org/berkeleylab/CheckV
Kraken 2	Wood et al.^14^	https://doi.org/10.5281/zenodo.3520272
Bracken	Lu et al.^15^	http://ccb.jhu.edu/software/bracken/
Decontam	Davis et al.^16^	https://github.com/benjjneb/decontam
MetaPhlAn 4	Blanco-Miguez et al.^17^	https://huttenhower.sph.harvard.edu/metaphlan/
Vegan	Oksanen et al.^18^	https://github.com/vegandevs/vegan/
glmmTMB	Brooks et al.^19^	https://cran.r-project.org/web/packages/glmmTMB/index.html
Emmeans	Searle et al.^20^	https://cran.r-project.org/web/packages/emmeans/index.html
MaAsLin	Mallick et al.^21^	http://huttenhower.sph.harvard.edu/maaslin2
mixOmics	Rohart et al.^22^	www.mixOmics.org
Opticlust	Westcott and Schloss^23^	https://github.com/SchlossLab/Westcott_OptiClust_mSphere_2017
RStudio	RStudio Team^24^	http://www.rstudio.com/

## Materials and equipment

**Table:** Pepsin solution

Reagent	Final concentration	Amount
Pepsin	4 mg/mL	0.04 g
Hydrochloric acid (1 M)	10 mM	0.01 mL
Nuclease free water		10 mL
**Total**		**10 mL**

Filter the solution using a 0.2 μm filter membrane. Store at 4°C for up to 12 months.

**CRITICAL:** Pepsin is a serious health hazard. It may cause genetic defects, damage to fertility or the unborn child, and allergy, asthma symptoms or breathing difficulties if inhaled. Hydrochloric acid is corrosive and may cause severe eye damage and skin burns on contact or splashing. Wear appropriate personal protective equipment and prepare pepsin solution in a fume hood.

**Table:** Norfloxacin solution

Reagent	Final concentration	Amount
Norfloxacin	1 mg/mL	0.01 g
Nuclease free water		10 mL
Sodium hydroxide solution (0.1 M)		A few drops to dissolve norfloxacin
**Total**		**10 mL**

Filter the solution using a 0.2 μm filter membrane. Store at 4°C for up to 12 months.

**Table:** Bottom agar

Reagent	Final concentration	Amount
LB broth (Miller)	25 g/L	5 g
Bacto dehydrated agar	1.5%	3 g
Distilled water		200 mL
**Total**		**200 mL**

Autoclave bottom agar at a temperature of 121°C and pressure of 15 psi for 20 min. Store the agar plates at 4°C for up to one month.

**Table:** Top agar

Reagent	Final concentration	Amount
LB broth (Miller)	25 g/L	2.5 g
Bacto dehydrated agar	0.4%	0.4 g
Calcium chloride (1 M)	0.001 M	0.1 mL
Distilled water		99.9 mL
**Total**		**100 mL**

Autoclave top agar at a temperature of 121°C and pressure of 15 psi for 20 min. Should be made fresh.

## Step-by-step method details

### Phage isolation and DNA extraction

**Timing: 7 h (per 12 reactions)**

**CRITICAL:** Ensure that all plasticware (tips and tubes) and solutions (media and buffers) used within the protocol are sterile and nuclease-free. All steps should be taken in a clean and sterile environment, ideally a class II microbiology safety cabinet to prevent contamination. We encourage incorporation of a negative control as part of the phage DNA extraction batch for checking and filtering potential contaminants.

1. Prepare phosphate buffered saline (PBS)-homogenized HM.
  a. Defrost bio-banked HM samples on ice.
    ***Note:*** For large volume study samples (≥ 50 mL), we recommend defrosting samples overnight in a fridge.
  b. Gently mix HM samples by inversion and pipette 200 μL of HM samples into a new 2 mL-microcentrifuge tube.
    **CRITICAL:** Gently mixing prior to pipetting is a crucial step because milk fat will settle at the top layer of samples over time after defrosting.
    ***Note:*** We recommend performing at least replicates per individual sample.
  c. Add 800 μL of 1x PBS solution and briefly vortex to homogenize samples.
    ***Note:*** We recommend homogenizing HM samples with 1x PBS solution in a ratio between 1:3 to 1:5. This step helps the separation of milk fat better. Researchers can also replace PBS solution with SM buffer.
2. Intense and horizontal agitation to disrupt the binding of microbes on the membrane of milk fat globules.^25^
  a. Vortex PBS-homogenized HM horizontally with maximum speed for 10 min.

**Figure 1:**
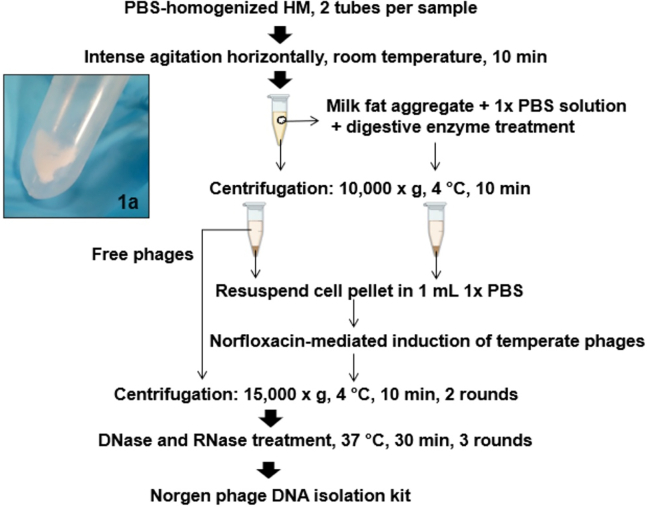
Optimized lytic and temperate phage isolation protocol for low volume preterm HM

***Note:*** This resulted in milk fat to aggregate and stick to the site of the tube (Figure 1A) for easier removal of milk fat in the next step. Researchers can increase the vortex time for larger volume samples.

3. Digestive enzyme treatment to hydrolyze milk fat.^26^
  a. Re-suspend milk fat aggregate obtained from step 2 in 800 μL of 1x PBS solution.
  b. Add 100 μL each of lipase (0.4 mg/mL) and pepsin (0.86 mg/mL) solution.
  c. Briefly vortex to mix the solution.
  d. Incubate at 37°C and shake at 370 rpm for 60 – 90 min.

***Note:*** This step is optional and will further release any microbes that are still binding to milk fat globule membrane after the agitation step.

4. Avoid touching the milk fat aggregate, pipette suspension (from steps 2 and 3) into a new 2 mL-microcentrifuge tube.
5. Centrifuge the suspension at a speed between 5,000 and 10,000 x g for 10 min at 4°C.

***Note:*** This step pellets eukaryotic and microbial cells. The remaining supernatant contains free viral particles. We use a lower speed in this step to pellet microbial cells for temperate phage induction. Researchers can use high speed to pellet and remove microbial cells if they are not interested in the study of temperate phages. Researchers can also increase centrifugation time for larger volume samples.

6. Avoid touching the cell pellet, pipette supernatant into a new 2 mL-microcentrifuge tube.

***Note:*** The supernatant contains free viral particles.

7. Norfloxacin-mediated induction of temperate phages.
  a. Re-suspend the cell pellet (from step 5) in 1 mL of 1x PBS solution.
  b. Add 1 μL of norfloxacin solution (0.001 mg/mL).
  c. Incubate at 37°C for 1 h.
  d. Centrifuge at 15,000 x g for 10 min at 4°C.
  e. Avoid touching the cell pellet, pipette supernatant into a new 2 mL-microcentrifuge tube.
  f. Repeat steps d and e.

***Note:*** This step is optional and only for researchers who wish to extract temperate phages. The supernatant contains induced temperate phages.

8. Pool both free viral and induced temperate phage supernatants obtained from steps 6 and 7 into a new 2 mL-microcentrifuge tube.
9. Nuclease-treatment to deplete extracellular nucleic acids.
  a. Add 2 μL of TURBO DNase and 1 μL of RNase Cocktail Enzyme Mix
  b. Incubate at 37°C for 30 min.
  c. Repeat steps a and b twice.
  d. Add 40 μL of 0.5 M EDTA solution (20 mM/mL) and incubate at 75°C for 10 min to deactivate DNase and RNase activities.

***Note:*** With the same final concentration of DNase (12U) and RNase (1.5 U) added, we found three rounds of 30 min-treatment is better than one-round of 1.5 h-treatment in depleting the extracellular nucleic acids in our samples, examined by the presence/absence of amplified bacterial gene fragments viewed on agarose gel electrophoresis.

10. Viral DNA extraction using Norgen Phage DNA Isolation Kit.

***Note:*** We follow the manufacturer’s protocol to extract viral DNA from nuclease-treated suspension. Researchers can use other commercial viral nucleic acid extraction kits or the conventional phenol-chloroform extraction method.

***Optional:*** Purify and concentrate viral DNA extracts using Agencourt AMPure XP beads (Beckman Coulter Genomics, USA) prior to sequencing.

### Single-phase lipidomic extraction for LC/MS-based discovery lipidomics

**Timing: up to 2 weeks (per 100 samples)**

11. Perform lipidomic extraction on ice and in a fume hood.
  a. Thaw HM samples on ice or in a fridge.
  b. Vortex samples for 10 seconds to homogenize samples.
  c. Pipette 100 μL of HM samples into a new 2 mL-Eppendorf LoBind tube.
    ***Note:*** We encourage researchers to work in a batch of 32 samples and include 2 extraction blanks for each extraction batch.
  d. Add 5 μL of SPLASH Lipidomix Quantitative mass spec internal standard to each sample.
  e. Add 1 mL of ice cold lipidomic extraction buffer (3:1 v/v Methanol and Dichloromethane) to each sample.
  f. Vortex for 10 seconds and sonicate in an ice water bath for 30 min.
  g. Centrifuge samples at 15,000 rpm for 15 min at 4°C.
  h. Transfer 900 μL of the supernatant into a new 2 mL-Eppendorf LoBind tube.
  i. Dry samples in a vacuum pre-concentrator at 45°C for 1.5 h.
    ***Note:*** The resulting lipid extracts can be stored at −80°C for up to 2 months.
12. Reconstitution of lipid extracts.
  a. Re-suspend extracted lipids in 200 μL of single-phase buffer (LC/MS grade water/Acetonitrile/isopropanol alcohol in 1:1:2 v/v).
  b. Sonicate for 15 min in an ice water bath.
  c. Centrifuge at 15,000 rpm for 15 min at 4°C.
  d. Transfer 190 μL of the supernatant to a 0.22 μm Costar spin filter.
  e. Centrifuge at 10,000 rpm for 5 min at 4°C.
  f. For a 1.5 mL autosampler vial with a 200 μL micro insert, transfer 80 μL of filtrate each for positive and negative mode profiling.
  g. Pool the remaining 30 μL together and vortex towards to the end as a quality control generation for data dependent acquisition.

***Note:*** We recommend creating one QC sample for every 10 samples. Any additional QC will be used for initial LC/MS system priming and stability assessment.

13. Prepare liquid chromatography buffer.
  ***Note:*** We conducted lipidomic separation using a Waters Acquity UPLC charge surface hybrid (CSH) Analytical column (2.1 mm x 100 mm with 1.7 μm stationary phase composition), Waters part number 180005297. We used Thermo scientific Vanquish Flex UHPLC system with a binary pump block set up for the analytical separation. We operated the LC Oven at 55°C with a flow rate of 200 μL/min.
  a. Prepare independent buffer systems for positive and negative mode polarity.
    i. Lipidomic Buffer A Positive: 1 L-buffer system (60% LC/MS Grade water/40% ACN) with 10 mM ammonium formate additives.
    ii. Lipidomic Buffer B Positive: 1 L-buffer system (90% IPA/10% ACN) with 10 mM ammonium formate additives.
      ***Note:*** Add 0.1% formic acid to both positive mode buffers prior to loading on the LC.
    iii. Prepare Lipidomic Buffer A and B Negative using the same composition as in steps i and ii, but without pH adjustment (neutral pH).
  b. Perform both positive and negative mode acquisition independently.
14. Wash the LC sample needle injector block assembly using rear seal wash (90% IPA and 5% ACN and 5 LC/MS grade with 0.1% formic acid).
15. Set LC gradient profile (Table 1).

**Table 1:** LC gradient profile of approximately 20 min injection to injection (curve 5)

T0	A (35%) / B (65%)
T1.5	A (35%) / B (65%)
T2.5	A (15%) / B (85%)
T8.5	A (1%) / B (99%)
T13.5	A (1%) / (99%)
T13.51	A (35%) / (65%)
18.5	A (35%) / B (65%)
16. Liquid Chromatography start up.
  a. Load both Positive buffers (A and B) onto LC system and installed LC line.
  b. Set the system to purge accordingly open purge valve on LC pump module.
  c. Set system to purge on the main instrumental plane.
    ***Note:*** This will take approximately 6 min; time may vary on different systems.
  d. Set flow to 100 μL/min with a buffer 50(A)/50(B) composition and LC oven to 55°C and allow for 20 min for system stabilization.
  e. Perform regular check for leaks to major connection points within the (1) LC to column, pump (2) block and (3) LC to MS connection within the LC module.
  f. Once the LC is up to the temperature and the analytical column has thermally stabilized, increase flow to 200 μL/min and wait for 10 min for the flow and pressure to stabilize.
  g. Continue to check for leaks.
  h. Allow the system to operate for 30 min and repeat steps e and f.
17. LC/MS system priming.
  a. Turn on HESI on the MS tune page, which will in turn bring the MS online.
  b. Set the flow rate on the HESI tune page interface to 200 μL/min.
  c. Apply voltage 3.5 kV (pos) and 2.5 kV (neg), respectively.
  d. Set sheath gas, aux gas, sweep gas, ion transfer tube temperature and vaporizer temperature.
    ***Note:*** Setting may vary between systems depending on internal performance requirement.
  e. Allow HESI and MS interface to thermally stabilize for a minimum of 30 min.
  f. Ensure no leak or change in pressure.
18. Perform MS1 and MS2 acquisitions.
19. Post acquisition.
  a. Cool down HESI for a minimum of 2 h with all gas flow and column flow rate on.
  b. Purge LC system with a 50/50 Aqueous ACN for a minimum of 3 h at 200 μL/min.
  c. Cool down ion transfer tube (ITT) temperature and wait for 1 h.
  d. Clean HESI blast shield and replace ITT before restarting the negative mode acquisition.

***Note:*** Negative mode acquisition is the same as the positive but with corresponding negative mode buffer and method file.

20. Check the LC/MS system while acquiring data (data generation, pressure profile, flow rate, leaks, contamination and QC response).
21. Data alignment.
  a. Convert all LC/MS raw data files into ABF format using ABF converter, see https://www.reifycs.com/AbfConverter/.
  b. Use MS-DIAL ver.4.00 software to align.

***Note:*** The alignment is for deconvolution, peak picking, alignment and compound identification. Parameter setting: MS1 tolerance, 0.005 Da/5 ppm; MS2 tolerance, 0.01 Da/10 ppm; minimum peak height: 25,000 amplitudes; mass slice width, 0.1 Da/100 ppm; smoothing method, linear weighted moving average; smoothing level, 5 scans; minimum peak width, 8 scans. [M + H]+, [M + NH 4]+, [M + Na]+, [2M + H]+, [2M + NH 4]+, [2 M + Na]+ were included in adduct ion setting for positive mode lipidomics and [M-H]–, [M + Cl]–, [M + Hac-H]– for negative mode.

22. Check the quality of peak table.
  a. Evaluate the analytical variation of the datasets using the peak table directly.
  b. Assess the QC profile and sum QC response.

***Note:*** The sum QC response across the entire analytical batch analysis should be 15% or less than any corresponding lipid features (ID or UNID) with RSD of 25% are retained and this extrapolated to the rest of the data set. Only stable signal is retained for downstream multivariate analysis.

### Bioinformatics and statistical analysis

**Timing: up to 9 days (per 100 samples)**

***Note:*** All data used in this protocol is publicly available at the European Nucleotide Archive under: PRJEB58774. This walkthrough produces no new programs or software. All are existing software is freely available to download and install as conda packages, see https://bioconda.github.io/conda-package_index.html.

**CRITICAL:** If Illumina sequencing was performed on the NextSeq, HiSeq or NovaSeq platforms, the reads for a single sample may be split across multiple lanes. This step is not required for MiSeq sequencing reads. Researchers need to concatenate these files prior to processing them. This can be done using the ‘concat_lanes.pl’ script available within Microbiome Helper, see https://github.com/LangilleLab/microbiome_helper/blob/master/concat_lanes.pl.

perl concat_lanes.pl ∗fastq.gz -o /path/to/desired/output/location

**CRITICAL:** Before and after each step in this protocol, it is important to produce a count of the sequence reads per sample for reporting. Commands below can be edited to run in any directory containing raw, processed or filtered reads. Once reads are converted from *fastq* to *fasta* format, change the script to count every second line, rather than every fourth.

23. Run commands in folders containing the raw (or concatenated) forward and reverse reads to print to total reads per sample as output by the sequencer.

# Count sequences per raw forward file #

find . -type f -name '∗R1_001.fastq' | while read -r i; do

 echo ${i##∗/}

 wc -l $i | awk '{print $1/4}' # count every 4^th^ line (fastq files have 4 lines per sequence

done > /path/to/desired/output/location/reads_per_fwd_fastq.tsv

# Reformat forward read count #

awk '{if(NR%2) a=$0; else print a"∖t"$0}' /path/to/previous/output/location/reads_per_fwd_fastq.tsv > /path/to/desired/output/location/formatted_reads_per_fwd_RAWfastq.tsv

# Count sequences per raw reverse file #

find . -type f -name '∗R2_001.fastq' | while read -r i; do

 echo ${i##∗/}

 wc -l $i | awk '{print $1/4}'

done > /path/to/desired/output/location/reads_per_rev_fastq.tsv

# Reformat reverse read count #

awk '{if(NR%2) a=$0; else print a"∖t"$0}' /path/to/previous/output/location reads_per_rev_fastq.tsv > /path/to/desired/output/location reformatted_reads_per_rev_RAWfastq.tsv

24. Quality filtering raw sequence reads.
  a. Perform FastP sequence quality filtering.
    i. Remove reads <100 nucleotides long, containing single base calls with phred score <20 or, starting at the 5′ end of the sequence and containing 5 nucleotide sliding windows with a mean phred score <25.
    ii. Place all QC filtered reads in a folder called ‘qc_reads’.
  b. Count sequences per forward and reverse read of each QC filtered sample in ‘qc_reads’ directory.
    # Make the output directory #
    mkdir /path/to/qc_reads
    # Apply the quality filter #
    for infile in ∗_R1.fastq
    do
    base=$(basename ${infile} _R1.fastq)
    fastp -i ${infile} -I ${base}_R2.fastq -o /path/to/qc_reads/${base}_R1.qc.fastq -O /path/to/qc_reads/${${base}_R2.qc.fastq --cut_front --cut_window_size 5 --cut_mean_quality 25 --qualified_quality_phred 20 --length_required 100 --thread 8 –h
    done
  c. Remove reads mapping to the human genome (GRCh37) from ‘qc_reads’ files with KneadData.
    ***Note:*** Where possible, we recommend subsetting all ‘qc_reads’ files into batches of max 10 samples within subdirectories and running parallel slurm scripts to reduce processing time.
    **CRITICAL:** Download the bowtie2 human indexes, see https://benlangmead.github.io/aws-indexes/bowtie. Providing full path to the database and the trimmomatic executable prevents errors.
    # Apply the human read filter #
    for infile in ∗_R1.qc.fastq
    do
    base=$(basename ${infile} _R1.qc.fastq)
    kneaddata –input ${infile} --input ${base}_R2.qc.fastq -db /full/path/to/download/location/bowtie_human --output . --trimmomatic /full/path/to/trimmomatic/executable/trimmomatic-0.39-2
    done
    # Make the output directory #
    mkdir /path/to/filter_reads
  d. Concatenate paired (overlapping) and unpaired (not-overlapping) human filtered reads per sample and then place the human filtered reads in a folder called ‘filter_reads’.
    # Concatenate the forward reads #
    for infile in ∗kneaddata_paired_1.fastq
    do
    base=$(basename ${infile} kneaddata_paired_1.fastq)
    cat ${infile} ${base}kneaddata_unmatched_1.fastq > /path/to/filter_reads/${base}kneaddata_1.fastq
    done
    # Concatenate the reverse reads #
    for infile in ∗kneaddata_paired_2.fastq
    do
    base=$(basename ${infile} kneaddata_paired_2.fastq)
    cat ${infile} ${base}kneaddata_unmatched_2.fastq > /path/to/filter_reads/${base}kneaddata_2.fastq
    done
  e. Count sequences per forward and reverse read of each human filtered sample in ‘filter_reads’ directory using example script above.
25. Assemble and assess contiguous sequences.
  a. Assemble contigs from ‘filtered_reads’ using Megahit.
    for file in ∗_1.fastq;
    do
    base=$(basename ${file} _1.fastq)
    megahit -1 "${base}"_1.fastq -2 "${base}"_2.fastq -o "${base}"_assembled.fasta/;
    done
  b. Extract assembled contigs for each sample from associated sample directory and then rename the file to include the sample provenance and place in directory ‘assembled’.
    # Make the output directory #
    mkdir /path/to/assembled
    # Find and extract the assemblies #
    find . -type f -name "final.contigs.fa" -printf "/%P∖n" | while read FILE ; do DIR=$(dirname "$FILE" ); cp ."$FILE" ."$DIR"_finalcontigs.fa;done
    find . -name "∗finalcontigs.fa" -exec cp {} ∼/path/to/assembled ∖;
  c. Count the number of contigs per sample in ‘assembled’ directory using modified example script above.
    ***Note:*** Remember to change from counting every fourth to every second line as contig files are in fasta, not fastq format.
    ***Optional:*** Researchers can use MetaQUAST to assess the general quality of metagenome assemblies prior to deduplication. Running MetaQUAST on raw assemblies would substantially increase the computational resources (time and memory). Instead, we use a custom approach including deduplication (step 25d), filtering assemblies on coverage (step 25f), identification of viral assemblies with VirSorter2 (step 25h) and quality checking with CheckV (step 25i).
  d. Deduplicate the contigs per sample using greedy clustering with CD-HIT-EST and then place the deduplicated contigs in directory ‘deduplicated’.
    ***Note:*** We chose to deduplicate based on 95% similarity of the largest (reference) contig across a minimum of 75% of the shorter (query) contig. Researchers can edit these parameters for specific use cases.
    # Make the output directory #
    mkdir /path/to/assembled/deduplicated
    # Run the deduplication #
    for infile in ∗.fa
    do
    base=$(basename ${infile} .finalcontigs.fa)
    cd-hit-est -i ${infile} -o deduplicated/${base}.dedupcontigs.fa -c 0.95 -aS 0.75 -T 0 -g 1 -M 0
    done
  e. Count the number of deduplicated contigs per sample in ‘deduplicated’ directory using the modified example script above.
  f. Map human filtered reads in ‘filter_reads’ directory on to the deduplicated contigs in ‘deduplicated’ directory.
    i. Make the deduplicated assembly index with BWA index.
    ii. Map filtered reads to deduplicated assemblies using BWA-MEM.
    iii. Extract read coverage statistics for each deduplicated assembly using BEDTools.
    iv. Extract only the assemblies with sufficient coverage with seqtk, see https://github.com/lh3/seqtk.
    v. Place the outputs in a new directory “filtered_contigs”.
      ***Note:*** This step quantifies read coverage of assembled contigs. In this study, we cull contigs with less than 2x coverage (minimum 2 reads mapped to each nucleotide). Researchers can change the contig coverage threshold for specific use cases. A lower coverage threshold increases the sensitivity of analyses to identify rare viral features, whilst a higher thresholds enables greater confidence in annotated contig quality.
      # Make the index (reference) files for each deduplicated assembly #
      for infile in /path/to/assembled/deduplicated/∗.dedupcontigs.fa
      do
      bwa index ${infile}
      done
      # Map filter_reads to deduplicated contigs #
      for infile in /path/to/assembled/deduplicated/∗.dedupcontigs.fa
      do
      base=$(basename ${infile}.dedupcontigs.fa)
      bwa mem ${infile} /path/to/filter_reads/${base}1.fastq /path/to/filter_reads /${base}2.fastq > ${base}.sam
      done
      # Convert .sam file to .bam #
      for infile in ∗.sam
      do
      base=$(basename ${infile} .sam)
      samtools view -h -b -S ${infile} > ${base}.bam
      done
      # Sort the .bam file #
      # Adjust the ‘-m’ flag to maximum memory capacity per node on your machine #
      for infile in ∗.bam
      do
      base=$(basename ${infile} .bam)
      samtools sort -m 1000000000 ${infile} -o ${base}.ordered.bam
      done
      # Extract contig coverage info #
      for infile in ∗.ordered.bam
      do
      base=$(basename ${infile} .ordered.bam)
      bedtools genomecov -ibam ${infile} -d -g lengths.genome > ${base}.bam.perbase.cov
      done
      # List only those contigs with >2x coverage #
      for infile in ∗.perbase.cov
      do
      base=$(basename ${infile} .bam.perbase.cov)
      awk -F"∖t" '$3>2{print $1}' ${infile} | sort | uniq -c > ${base}.perbase.count
      done
      # Keep only the second column containing contig IDs #
      for infile in ∗.perbase.count
      do
      base=$(basename ${infile} .perbase.count)
      awk '{print $2}' ${infile} > ${base}.contigs.keep
      done
      # Create new filtered file containing only the contigs with >2x coverage #
      for infile in ∗dedupcontigs.fa
      do
      base=$(basename ${infile} .dedupcontigs.fa)
      seqtk subseq ${infile} ${base}.contigs.keep > ${base}.2x_filtered.contigs.fa
      done
      # Move the filtered contigs files to new directory #
      mkdir /path/to/filtered_contigs/
      cp ∗2x_filtered.contigs.fa /path/to/filtered_contigs/
  g. Count the number of contigs with 2x coverage per sample in ‘filtered_contigs’ directory using modified example script above.
  h. Identify viral contigs (including dsDNA, ssDNA, RNA and Nucelocytoviricota) from 2x filtered contigs in ‘filtered_contigs’ directory using VirSorter2.
    ***Note:*** Researchers can change the ‘–include-groups’ flag to exclude certain viral types according to specific research question and upstream processing.
    **CRITICAL:** Download and install the VirSorter database prior to running, see https://github.com/jiarong/VirSorter2#download-database-and-dependencies.
    for infile in /path/to/filtered_contigs/∗.fa
    do
    base=$(basename ${infile} .2x_filtered.contigs.fa)
    virsorter run --keep-original-seq -w ${base}.virsort -i ${infile} --include-groups dsDNAphage,NCLDV,RNA,ssDNA -j 4 all
    done
  i. Predict quality of viral contigs and viral lifecycle using CheckV.
    ***Note:*** Researchers who are interested in assembling whole viral genomes from metagenomes should check the output of this step for contigs annotated as high-quality or complete by CheckV.
    **CRITICAL:** Download and install the CheckV database prior to running, see https://bitbucket.org/berkeleylab/checkv/src/master/. The full path to location of database is given with ‘-d’ flag when running ‘checkv’ command.
  j. Count the number of viral, proviral and total virus contigs per sample.

# Make the output directory #

mkdir /path/to/viral_contigs

# Calculate filtered contig completeness and quality #

for infile in /path/to/filtered_contigs/∗.virsort/final-viral-combined.fa

do

base=$(basename ${infile} .virsort/final-viral-combined.fa)

checkv end_to_end ${infile} /path/to/viral_contigs/${infile}_checkV -d /path/to/checkv-db-v1.0

done

# Rename per-sample directories so can extract proviral and viral confirmed sequences from subdirectories without losing sample traceability #

for i in /path/to/filtered_contigs/∗virsort; do echo -v "$i" $(echo $i | sed 's/∖virsort//g'); done

# Extract confirmed proviral sequences from sub directories #

for DIR in /path/to/filtered_contigs/∗

do

 test -d "$DIR" -a -f "$DIR/final-viral-combined.fa_checkV/proviruses.fna" || continue

 mv -f "$DIR/final-viral-combined.fa_checkV/proviruses.fna" "$DIR.proviruses.fna"

done

# Extract confirmed viral sequences from sub directories #

for DIR in /path/to/filtered_contigs/∗

do

 test -d "$DIR" -a -f "$DIR/final-viral-combined.fa_checkV/viruses.fna" || continue

 mv -f "$DIR/final-viral-combined.fa_checkV/viruses.fna" "$DIR.viruses.fna"

done

# Researchers may wish to remove needless subdirectories now the proviral and firal .fna files have been extracted. If so, remove the hash at the beginning of the next line #

# rm -rf ./∗/

# Concatenate proviral and viral .fna files together for each sample. Results in single file <sample>.index.fna #

for i in /path/to/filtered_contigs/∗.proviruses.fna ; do

base=$(basename ${i} .proviruses.fna)

cat ${i} ${base}.viruses.fna > /path/to/viral_contigs/${base}.index.fna

done

26. Predict and annotate genes on viral contigs in ‘viral_contigs’ directory using VIBRANT.

***Note:*** The formatted output generated here contains only auxiliary metabolic gene status (y/n), KO, Pfam and VOG annotations of each gene per viral contig per sample. Running VIBRANT produces far more information. For full description of VIBRANT output, see https://github.com/AnantharamanLab/VIBRANT/blob/master/output_explanations.pdf.

**CRITICAL:** Download and install the VIBRANT database prior to running, see https://github.com/AnantharamanLab/VIBRANT.

# Make the output directories #

mkdir /path/to/annotation

mkdir /path/to/annotation/functional

# Classify viral contigs with VIBRANT #

for infile in /path/tp/viral_contigs/∗index.fna

do

base=$(basename ${infile} index.fna)

VIBRANT_run.py -i ${infile} -folder /path/to/annotation/functional -virome;

done

# Extract vibrant functional gene annotations to new file suffixed ‘formatted_index.tsv’ #

find /path/to/annotation/functional/ -name "VIBRANT_annotations∗" -exec cp {} /path/to/annotation/functional/ ∖;

for infile in /path/to/annotation/functional/∗.tsv

do

base=$(basename ${infile} .index.tsv)

cut -f1,4,5,10,15 ${infile} > /path/to/annotation/functional/${base}.formatted_index.tsv

done

27. Quantify viral contigs within each sample.
  a. Count total number of reads mapping to each viral contig per sample.
    ***Note:*** Output ‘∗contig_rawFREQ.tsv’ will contain 7 columns detailing: 1 - Contig name; 2 - start pos (bp); 3 - end pos (bp); 4 - raw read frequency; 5 - read mapping length; 6 - total contig length; 7 - percent of contig covered by mapped reads.
    # Create the mapping index for viral_contigs in each sample #
    for infile in /path/to/viral_contigs/∗index.fna
    do
    bwa index ${infile}
    done
    # Map human filtered sequence reads on to viral contigs using BWA-MEM #
    for infile in /path/to/viral_contigs/∗index.fna
    do
    base=$(basename ${infile} .index.fna)
    bwa mem ${infile} /path/to/filtered_reads/${base}_1.fastq /path/to/filtered_reads/${base}_2.fastq > ${base}.sam
    done
    # Convert .sam file to .bam #
    for infile in /path/to/viral_contigs/∗.sam
    do
    base=$(basename ${infile} .sam)
    samtools view -h -b -S ${infile} > /path/to/viral_contigs/${base}.bam
    done
    # Sort the .bam file #
    for infile in /path/to/viral_contigs/∗.bam
    do
    base=$(basename ${infile} .bam)
    samtools sort -m 1000000000 ${infile} -o /path/to/viral_contigs/${base}.ordered.bam
    done
    # Extract raw contig statistics (sequence reads mapping to viral contigs) #
    for infile in /path/to/viral_contigs/∗.ordered.bam
    do
    base=$(basename ${infile} .ordered.bam)
    bedtools genomecov -bg -ibam ${infile} | bedtools merge -i stdin | bedtools coverage -a stdin -b ${infile} > /path/to/viral_contigs/${base}.contig_RAWfreq.tsv
    done
  b. Format the read count mapping file to normalize for contig length and read depth per sample using R script ‘RPM_Normalize.R’.
    # Make the output directory #
    mkdir /path/to/Normalised_freqs/
    ## RPM_Normalize.R ##
    # Get a list of files #
    input_files <- list.files(path = "path/to/your/output/directory" , pattern = "[.]contig_RAWfreq.tsv")
    # Loop for reading input and writing an output #
    for(i in 1:length(input_files)){
     filename <- input_files[i]
     df0 <- read.delim(input_files[i], header = F)
     colnames(df0) = c("Contig_ID", "start", "end", "raw_reads", "reads_over", "length", "pc_coverage")
     df0$Reads_per_kb = (df0$raw_reads/(df0$reads_over/1000))
     df0$Freq_per_mil_reads = (df0$Reads_per_kb/(sum(df0$raw_reads)/1000000))
     write.table(df0, file = file.path("path/to/new/output/directory", paste0(filename,"_output.tsv")), sep = '∖t', col.names = T, row.names = F)
    }
  c. Combine the per-sample normalized frequency tables to produce a single table containing all samples using R script ‘Merge_counts.R’.

# Extract the important information from the per-sample tables #

for I in ∗tsv_output.tsv; do J=$( basename $I .contig_RAWfreq.tsv_output.tsv ); cut -f1,9 ${I} | sed "s/Freq_per_mil_reads/${J}/" > ${J}.freq.mil.tsv ; done

## Rscript Merge_counts.R ##

install.packages('tidyverse', repos = "https://cloud.r-project.org")

install.packages('dplyr', repos = "https://cloud.r-project.org")

library(tidyverse)

library(dplyr)

# Get a list of files #

input_files <- list.files(path = "path/to/your/output/directory" , pattern = "[.]freq.mil[.]tsv")

# Read files into one big list #

myfiles = lapply(input_files, read.table, header = TRUE)

# Apply meaningful names to list entries (originating files) #

df <- Reduce(full_join, myfiles)

write.table(df, file = "path/to/new/output/directory", sep = ',')

28. Taxonomic annotation of viral contigs.
  a. Annotate viral contigs in ‘viral_contigs’ directory using kmer sequence similarity to pre-built NCBI viral database with Kraken2.
    **CRITICAL:** Download the Kraken2 database, see https://benlangmead.github.io/aws-indexes/k2.
    # Make the output directories #
    mkdir /path/to/annotation/taxonomy
    for infile in /path/to/viral/contigs/∗.index.fna
    do
    base=$(basename ${infile} .index.fna)
    kraken2 --use-names --threads 24 --report ${base}.kreport --report-zero-counts --db /path/to/database/k2_viral_20210517 ${infile} --output /path/to/annotation/taxonomy/${base}.kraken --unclassified-out /path/to/annotation/taxonomy/${base}.uncl
    done
  b. Calculate relative abundance of each taxonomic classification using bracken.
    for infile in /path/to/annotation/taxonomy/∗.kreport
    do
    base=$(basename ${infile} .kreport)
    bracken -d path/to/database/k2_viral_20210517 -i ${infile} -o /path/to/annotation/taxonomy/${base}.bracken_S -l S -t 1
    done
  c. Merge per-sample bracken output tables using combine_kreports.py script available at kraken_tools, see https://github.com/jenniferlu717/Bracken/tree/master.
    /path/to/script/combine_braken_outputs.py --files /path/to/annotation/taxonomy/∗.bracken_S -o /path/to/annotation/taxonomy/merged_bracken_species.tsv
  d. Infer viral host genus of closest genomic annotation (as per kraken) manually by searching the taxonomic annotation in the NCBI Taxonomy Browser.
    ***Note:*** Since this method was utilized more efficient (and more accurately) ways to predict bacteriophage host range have been developed. These host range prediction tools often employ machine learning models based on sequence data. While their accuracy may be greater for whole assembled bacteriophage genomes their utility in predicting host range in metagenomically assembled contigs, hence the approach employed here. As these tools develop their utility will undoubtedly increase.
29. Quantification and statistical analysis using RStudio.
  **CRITICAL:** Download and install both R and RStudio, see https://rstudio-education.github.io/hopr/starting.html
  a. Import count, taxonomy and associated metadata tables, and then convert to phyloseq formatted otu_tables, tax_tables and sample_data tables as outlined in phyloseq tutorial, see https://joey711.github.io/phyloseq/import-data.html.
  b. Perform Fisher’s exact test to test differences in any categorical variables of metadata between samples included analysis groups (Week of lactation).
    # Do the test #
    Fisher_CoVariate1 = fisher.test(METADATA$CoVariate1, METADATA$Lactation_week)
    # View results #
    Fisher_CoVariate1
  c. Perform Kruskal-Walis rank sum test to test hypothesis that differences in any continuous variables of metadata exist between analysis groups.
    # Do the test #
    KW_CoVariate1 = kruskal.test(METADATA$CoVariateA ∼ METADATA$Lactation_week)
    # View results
    KW_CoVariate1
  d. Calculate and plot viral contig presence per sample.
    # Calculate library size #
    METADATA$LibSize = sample_sums(Raw_phyloseq_object)
    # Order the metadata table by library size #
    METADATA = METADATA [order(METADATA $LibSize),]
    # Assign an index value to each row #
    METADATA$Index = seq(nrow(METADATA))
    # Define a colour pallete for plotting #
    sample.cols = c('Control' = "grey20", 'Sample' = "goldenrod")
    # Plot contig presence in samples from the lowest to highest number #
    ggplot(data = METADATA) + geom_point(aes(x=Index, y=LibSize, fill = Sample_control), size = 4, stroke = 1, shape = 21) +
     theme_classic2() + scale_y_log10(name = "Library size") + guides(alpha = "none", fill = guide_legend(title="Library")) +
     ggtitle("Library size", subtitle = "2x contigs") +
    scale_fill_manual(values = sample.cols) +
     theme(legend.position = "bottom",
     plot.title = element_text(size = 20, face = 'bold'),
     plot.subtitle = element_text(size = 13, face = 'bold'),
     legend.text = element_text(size = 15, face = 'bold'),
     legend.title = element_text(size = 15, face = 'bold'),
     axis.text = element_text(size = 15, face = 'bold'),
     axis.title = element_text(size = 15, face = 'bold'))
  e. Examine possible contamination and confirm samples suitable to address research questions.
    i. Compare library size between samples and negative controls using Kruskal Wallis test.
    ii. Compare dissimilarity between community compositions using ANOSIM based on Bray-Curtis dissimilarity.
      ***Note:*** Samples have a significantly greater library size and are significantly dissimilar in community compositions as compared to negative controls.
      # Perform kruskal test on library sizes #
      library.test = kruskal.test(METADATA$LibSize ∼ METADATA$Sample_Control)
      # View results #
      library.test
      # Plot the boxplot to show different library sizes #
      library.ctrl = ggplot(samples.size, aes(x = sample, y = library)) +
       #geom_boxplot(aes(fill = sample), colour = c("black"), outlier.shape = NULL) +
       geom_jitter(aes(fill=sample), colour = c("black"),
       size = 3, shape = 21, stroke = 1, width = 0.1, height = 0) +
       scale_y_continuous(name = "Library size∖n (classified contigs)") +
       ggtitle(label = "Library size comparison", subtitle = "2x contigs") +
       theme_classic2() +
       scale_fill_manual(values = sample.cols) +
       scale_colour_manual(values = sample.cols) +
       theme(legend.position = "NULL",
       axis.title.x = element_blank(),
       axis.text = element_text(size = 15, face = 'bold'),
       axis.title = element_text(size = 15, face = 'bold'),
       plot.title = element_text(face = 'bold', size = 18),
       plot.subtitle = element_text(face = 'bold', size = 13))
      # View the plot #
      library.ctrl
      # Extract feature table from phyloseq object #
      compareOTU = otu_table(Raw_phyloseq_object)
      # Calculate Bray-Curtis dissimilarity between samples #
      CtrlVSam = vegdist(t(compareOTU), method="bray")
      CtrlVSam = as.matrix(CtrlVSam) # Convert dissimilarity to matrix
      CtrlVSam = as.data.frame(CtrlVSam) # Convert to data frame
      # Extract sample/control grouping variable from phyloseq object #
      CvS = sample_data(Raw_phyloseq_object)$Sample_control
      # Perform ANOSIM test #
      anosim(CtrlVSam, CvS)
  f. Cull any potential contaminant taxa from sample feature table using a strict probability threshold (50%) and the prevalence method in negative control samples as defined by the decontam package.
    ***Note:*** Researchers can manipulate this threshold to increase the sensitivity to contaminants for removal (increasing threshold) or reduce sensitivity to maximize specificity to contaminants for removal (reducing threshold). This will depend largely on the sample of interest. For relatively low biomass samples (like those included in this study), we recommend a conservative approach to contaminant identification and removal.
    i. Plot correlation between prevalence in samples and controls to infer transfer of taxa reads from negative control to sample and visa-versa.
    ii. Manually inspect taxa identified as potential contaminants.
      ***Note:*** Features identified as (and not as) contaminants may not always make biological sense. Researchers should exercise caution in accepting decontam results without curation.
      # Identify contaminants based on prevalence in negative controls #
      sample_data(Raw_phyloseq_object)$is.neg = sample_data(Raw_phyloseq_object)$Sample_control == "Control"
      contamdf.prev = isContaminant(Raw_phyloseq_object, method = "prevalence", neg = "is.neg", threshold = 0.5)
      table(contamdf.prev$contaminant) # Tabulate potential contaminant taxa
      contamdf.prev$Species = compareTAX$Species # Assign taxonomic annotation from pyloseq object
      # View which taxa are potentially sequence negative contaminants #
      which(contamdf.prev$contaminant)
      ## CONFIRM LEGITIMACY OF IDd CONTAMINANTS ##
      #Export the negatives as separate phyloseq #
      Controls = subset_samples(Raw_phyloseq_object, sample_data(Raw_phyloseq_object)$Sample_control == "Control")
      #EXPORT THE POSITIVES AS SEPERATE PHYLOSEQ
      Samples = subset_samples(Raw_phyloseq_object, sample_data(Raw_phyloseq_object)$Sample_control == "Sample")
      ## Create object of presence/absence in seq controls
      Controls.presence = transform_sample_counts(Controls, function(abund) 1∗(abund>0))
      # Create object of presence/absence in biological samples #
      Samples.presence = transform_sample_counts(Samples, function(abund) 1∗(abund>0))
      # Create data.frame of taxon prevalence in positive/negatives #
      df.pres = data.frame(prevalence.pos = taxa_sums(Samples.presence),
       prevalence.neg = taxa_sums(Controls.presence),
       contam.prev = contamdf.prev$contaminant)
      # Perform the linear regression to check significance of relationship between prevalence of sample/controls #
      PREV.mod = lm(df.pres$prevalence.neg ∼ df.pres$prevalence.pos, data = df.pres)
      summary(PREV.mod) # View results (often any reads in negative controls have crossed over from highly abundant samples therefore expect a strong positive correlation between sample and control prevalence)
      # Plot scatter of taxon abundance in positive/negatives. First must convert prevalences to numerics to allow log(lm) function #
      ggplot(data=df.pres, aes(x=prevalence.neg, y=prevalence.pos, alpha=0.8, colour=contam.prev)) +
       geom_point(show.legend=F) +theme_classic2() +
       geom_text(aes(label=row.names(otu_table(combine))),colour="black", alpha=0.5) +
       geom_smooth(method=lm, inherit.aes = TRUE, alpha=.3, size=0.2, fullrange=T, show.legend=F, colour='black', lty='dashed')
      # Manually inspect the scatter plot to identify any features that are likely contaminants:
      # A - more prevalent in controls than samples
      # B – Not following prevalence trend between samples & controls
      # C – Borderline on prevalence threshold but non biologically sensible in system #
      # Mark taxa for clearance #
      ContamTax = subset.data.frame(df.pres, df.pres$contam.prev=='TRUE') # define taxon prevalent in controls
      ContamTax = rownames(ContamTax)
      ContamTax = c("VTU0026","VTU0027","VTU0032","VTU0085","VTU0128","VTU0161","VTU0172","VTU0173",
       "VTU0190","VTU0193","VTU0213","VTU0227","VTU0232","VTU0230")
      KeepTax <- setdiff(taxa_names(Raw_phyloseq_object), ContamTax) # select all other taxa to keep
      Analysis_phyloseq <- prune_taxa(KeepTax, Raw_phloseq_object) # create new phyloseq object containing just good taxa
      taxa_names(Analysis_phyloseq) # check bad taxa have been removed
  g. Re-check library sizes and community dissimilarity between samples and controls using edited version of script form step 27 above.
    ***Note:*** Sample library sizes should not have been substantially reduced, whilst negative and kit control library sizes should be.
  h. Agglomerate taxonomic levels and normalize feature counts for analysis.
    ***Note:*** All examples provided below are at the ‘Genus’ taxonomic rank. Researchers can substitute variables for the desired taxonomic rank of analysis (e.g. Family, Species, etc.).
  i. Combine individual features at each of the taxonomic ranks to analyze.
    ***Note:*** As the deepest taxonomic rank, no agglomeration is required at species level.
    # Genus level agglomeration #
    Genus = tax_glom(Analysis_phyloseq, taxrank = "Genus")
  j. Re-normalize the agglomerated taxonomic rank phyloseq objects by recalculating summed relative abundances.
    # Genus level normalization #
    Genus_perc = transform_sample_counts(Genus, function(x) 100 ∗ x/sum(x))
  k. Produce heatmap of top 20 most abundant taxonomic features to visualize overall community structure.
    ***Note:*** The total number of features at deeper taxonomic ranks will be much greater than 20. In such cases it is important to balance the number of features to include on heatmap for visualization purposes with loss of substantial proportions of taxa contribution to overall community structure. Assessment of this can be achieved by using the sample sums() function before and after filtering taxa to identify optimum number of features to include in the heatmap.
    ## Genus heatmap ##
    # Subset the top 20 most abundant taxa #
    Genus_heat = prune_taxa(names(sort(taxa_sums(Genus_perc),TRUE)[1:50]), Genus_perc)
    Genus_heatmap = plot_heatmap(Genus_heat, sample.label = "Lactation_week", taxa.label = "Genus", sample.order = "Lactation_week",
     low = "#FEE5D9", high = "#A50F15", na.value = "white", trans = log_trans(2),
     title = "Viral Genus", first.sample = "10A_S73") +
     theme_classic2() +
     theme(axis.title.y = element_blank(),
     axis.title.x = element_blank(),
     axis.text.x = element_text(angle = 90),
     axis.text.y = element_text(hjust = 1, angle = 25, face = 'bold'),
     plot.title = element_text(size = 15, face = 'bold'),
     plot.margin = unit(c(5,0,50,5), "pt"))
    Genus_heatmap
  l. Identify core bacteriophage features at each taxonomic rank. Test for significant positive correlations between relative abundance and distribution of bacteriophage features across samples, using a cut-off of 50% distribution to identify the core features.
    # Define a function to calculate the sample distribution of features #
    # Returns numbers of non-zero values in a row #
    distribution_count <- function(x){sum(x > 0)}
    # Create the DAR table #
    # Contains three columns, feature name (taxonomy), percent distribution and mean abundance #
    DAR_table = data.frame(feature = Taxonomy_table$Genus,
     distribution = (apply(Genus_otu_table, 1, disribution_count)/ncol(Genus_otu_table))∗100,
     abundance = (rowMeans(Genus_otu_table)))
    # Compute the linear regression statistic #
    DAR.mod = lm(prevalence ∼ abundance, data = DAR_table)
    # View results #
    summary(DAR.mod)
    # Significant P value and large adjusted R2 values indicate strong correlation #
    # Plot the distribution abundance relationship #
    DAR = ggplot(data = DAR_table) +
     geom_point(x=DAR_table$distribution, y=DAR_table$abundance, fill = c("#A50F15"), colour = c("black"), shape = 21, size = 3) +
     geom_smooth(aes(x=DAR_table$distribution, y=DAR_table$abundance), colour = c('#A50F15'), fill = c('#A50F15'), stat = 'smooth', lty = 'dashed', fullrange = T, alpha = 0.5) +
     scale_x_continuous(name = "Distribution (%)") +
     scale_y_continuous(name = "Mean Relative Abundance") +
     ggtitle("Genus", subtitle = "Overall (n = 99)") + # change subtitle depending on sample number included in analysis
     theme_classic2() +
     theme(legend.position = "none",
     axis.text = element_text(face = 'bold', size = 25),
     axis.title = element_text(face = 'bold', size = 25),
     plot.title = element_text(face = 'bold', size = 30),
     plot.subtitle = element_text(face = 'bold', size = 20))
    DAR
  m. Compare the core features of the phageome at each taxonomic rank overall, and at each lactation stage.
    i. Use Bonferroni correction to account for multiple hypothesis testing and report taxa with >50% sample distribution as core.
    ii. Calculate rarefied viral taxonomic richness and viral Shannon diversity across each taxonomic rank.
      ***Note:*** Richness calculation requires integers; therefore, it is calculated using rarefied raw read counts. Shannon diversity calculation does not require integers, therefore it is calculated using proportionally normalized read counts, to avoid unnecessarily discarding data points.
      # Define lowest sample read depth for rarefaction #
      summary(sample_sums(Genus_reads_phyloseq)
      # Select round value below minimum sample depth for rarefaction. If minimum sampling depth is not suitable for analysis, consider discarding samples rather than rarefying to such a low depth #
      # Calculate the rarefied viral richness #
      METADATA$Richness = rarefy(t(Genus_reads_otu_table), 400)
      # Calculate the viral Shannon diversity #
      METADATA$Shannon = diversity(t(Genus_reads_otu_perc), index = "shannon")
  n. Analyze the relationship between viral richness and diversity with lactational stage at each taxonomic rank using generalized mixed linear models and include covariates in the model corrects for confounding impact of other relevant variables on measures of alpha diversity.
    **CRITICAL:** Check the distribution of data with a histogram to inform model parameterization. Once the model is built check the Akaike Information Criterion (AIC) for goodness-of-fit before performing a statistical test on the variable of interest (Lactation stage). A lower AIC = a better model.
    ***Note:*** Example below demonstrates building a model to test the relationship between the lactation stage and taxonomic richness. Variables can be edited to swap taxonomic richness for Shannon diversity.
    # Summarize the Richness values #
    summary(METADATA$Richness)
    # Plot a histogram to check the distribution of data #
    ggplot(METADATA, aes(Richness)) +
     geom_histogram(alpha=.8, colour = "black") +
     facet_wrap(∼Lactation_week, nrow = 1)
    # Build the glm #
    summary(RA.genus =
     glmmTMB(Richness ∼ Lactation_week + GA_weeks + Delivery_mode + (1|Patient_no), data = METADATA, family = poisson()))
    testResiduals(RA.genus) # check model fit (AIC) & residuals.
    car::Anova(RA.genus) # test influence of covariates on outcome measure
    # Run Post-hoc, pairwise tests #
    RAgen.Lweek = emmeans(RA.genus, specs = pairwise ∼ Lactation_week)
    # Plot the relationship between richness and lactation stage #
    Lactation.gen_rich = ggplot(METADATA, aes(x = Lactation_week, y = Richness)) +
     geom_violin(aes(fill = Lactation_week), colour = c("black"), size = 1) +
     geom_jitter(aes(fill = Lactation_week), colour = c("black"), width = 0.2, height = 0, size = 3, shape = 21, stroke = 1) +
     scale_fill_brewer(palette = "Reds") + theme_classic2() +
     xlab("Lactation age") +
     ylab("Taxonomic Richness") +
     ggtitle("Genus") +
     theme(legend.position = "none",
     plot.title = element_text(size = 20, face = 'bold'),
     legend.text = element_text(size = 15, face = 'bold'),
     legend.title = element_text(size = 15, face = 'bold'),
     axis.text = element_text(size = 15, face = 'bold'),
     axis.title = element_text(size = 15, face = 'bold'))
  o. Calculate Bray-Curtis dissimilarity between samples and use PERMANOVA to identify longitudinal shifts in phage community compositions across each taxonomic rank.
    # Calculate Bray-Curtis dissimilarity #
    bc.G = vegdist(t(otu_table(Genus_reads_otu_perc)), method = "bray")
    bc.G = as.matrix(bc.G) # Convert dissimilarity matrix to matrix
    bc.G = as.data.frame(bc.G) # Convert matrix to data frame
    # Perform the PERMANOVA #
    G.adonis = adonis2(bc.G ∼ Lactation_age, data = METADATA, by = 'margin')
    # Compute principle components #
    pca.G <- prcomp(bc.G[,-1], center=TRUE, scale=TRUE)
    # Draw the biplot #
    PCA.G = ggbiplot(pca.G, choices=(1:2),
     var.axes=F, obs.scale =.2,
     ellipse=T, alpha = 0, groups = Lactation_age, colour = Lactation_age) +
     geom_point(aes(fill=Lactation_age, size=5, stroke=1), shape=21,) +
     scale_fill_brewer(palette = "Reds") + scale_colour_brewer(palette = "Reds") +
     theme_classic2() +
    ## Custom script to plot group centroids on PCA ##
    # Extract the euclidean locations #
    G_w_dists = as.data.frame(pca.G$x)
    # Assign the variables to table #
    G_w_dists$Lactation_age = METADATA$Lactation_age
    # Calculate the centroid location #
    G_w_centroid.LA = merge(G_w_dists,aggregate(cbind(mean.PC1=PC1,mean.PC2=PC2) ∼ Lactation_age,G_w_dists,mean),by="Lactation_age")
    # Plot the new PCA #
    PCA.G_w_centroid.LA = ggplot(G_w_centroid.LA, aes(PC1,PC2)) +
     geom_point(aes(fill = Lactation_age), size=3, shape = 21) +
     scale_fill_brewer(palette = "Reds") +
     scale_colour_brewer(palette = "Reds") +
     geom_point(aes(x=mean.PC1,y=mean.PC2, fill = Lactation_age),size=6, stroke = 1, shape = 21) +
     geom_segment(aes(x=mean.PC1, y=mean.PC2, xend=PC1, yend=PC2, colour = Lactation_age), alpha = 0.7) +
     xlab("PC1 (38.0% variance)") + ylab("PC2 (17.4% variance)") +
     theme_classic2() + ggtitle("Genus") +
     theme(axis.title = element_text(size=20, face = 'bold'),
     axis.text= element_text(size=20, face = 'bold'),
     legend.position="bottom",
     legend.text = element_text(size=18, face = 'bold'),
     legend.title = element_text(size=20, face = 'bold'),
     legend.direction = 'vertical', plot.title = element_text(face = "bold", size = 22)) +
     guides(groups=guide_legend("Lactation age", shape=FALSE, size=FALSE))
  p. Identify features of the phageome that are present as significantly different abundances across lactation stage at each taxonomic rank using MaAsLin2.
    i. Set minimum abundance and prevalence thresholds of 10% to ensure only taxa present at enough to influence biology of the system are identified.
    ii. Arcsine squared transform proportional feature counts.
      **CRITICAL:** Researchers must set the directory variable to a location in the file system that they have access to. This is where MaAsLin2 will print all outcome data.
      setwd("/path/to/desired/results/output/directory")
      G.Maaslin_results <-
       Maaslin2(input_data = Genus_perc,
       input_metadata = METADATA,
       output = "Maaslin_genus",
       fixed_effects = c('Lactation_age_day','GA_weeks','Delivery_mode'),
       transform = "AST",
       reference = 'Delivery_mode,Vaginal',
       plot_scatter = TRUE,
       min_abundance = 0.1,
       min_prevalence = 0.1,
       cores = 4)
  q. Manually inspect file ‘all_results.tsv’ in MaAsLin2 output directory for each taxonomic rank.
  r. Combine rows related to the “Lactation_age_day” variable to create one large table detailing relationship of the variable of interest to proportional abundance for each taxonomic rank.
  s. Re-import manually curated MaAsLin2 results table then produce differential abundance plot.

# Import the data #

all_maas = read.csv(“/path/to/combined/MaAsLin2/results.csv”, header = T, row.names = 1)

# Define the colour palette #

Analysis_cols = c("Viral species" = "#238B45", "Viral host" = "#74C476", "Viral genus" = "#BAE4B3", "Viral family" = "#EDF8E9")

# Construct the plot object #

Maas_plot = ggplot(all_maas, aes(x = value, y = trans_coef)) +

 geom_jitter(data = all_maas, alpha = 0.3, size = 3, width = 0.3, height = 0, shape = 21, stroke = 1, aes(fill = Analysis, colour = Analysis)) +

 geom_jitter(data = sig_maas, size = sig_maas$Size, width = 0.3, height = 0, aes(fill = Analysis), colour = "firebrick", shape = 21, stroke = 1) +

 scale_colour_manual(values = Analysis_cols) + scale_fill_manual(values = Analysis_cols) +

 geom_abline(slope=0, intercept=0, linetype = "dashed", colour = "grey30") +

 theme_classic2() + ggtitle("Proportional abundance compared to mature milk") +

 guides(fill = guide_legend(nrow = 2, byrow = T), colour = "none") +

 labs(x = "Lactation age", y = 'Maaslin coefficient∖n(cube root transformed)') +

 theme(legend.position = "bottom",

 legend.text = element_text(size=13, face = "bold"),

 legend.title = element_blank(),

 legend.direction = "horizontal",

 plot.margin = margin(t=15, r=15, b=0, l=10, unit = "pt"),

 axis.title.x = element_text(size=18, face = "bold"),

 axis.title.y = element_text(size=18, face = "bold"),

 axis.text.x = element_text(size=15, face = "bold"),

 axis.text.y = element_text(size=15, face = "bold"),

 plot.title = element_text(face = 'bold', size = 18, hjust = 0)) +

Maas_plot

### *In vitro* phage-lipid assay

**Timing: 2 days**

**CRITICAL:** Ensure that all plasticware (tips and tubes) and solutions (media and buffers) used within the protocol are sterile. All steps should be taken in a clean and sterile environment to prevent contamination.

30. Prepare the phage host *Escherichia coli* (*E. coli*) culture for plaque assay.
  a. On the day before *in vitro* phage-lipid assay, inoculate *E. coli* into a universal bottle containing 10 mL of LB broth.
  b. Incubate at 37°C and shake at 200 rpm overnight (16–18 h).
  c. Pipette 100 μL of overnight *E. coli* culture into a new universal bottle containing 10 mL of LB broth.
  d. Incubate at 37°C and shake at 200 rpm for about 3 h.

***Note:*** This resulted in exponential growth of *E. coli* with an OD600 density reading value of 0.5–0.6.

31. Perform phage-lipid incubation.
  a. Prepare 12 reactions of *E. coli* phage with and without linoleic acid in a 1.5 mL-microcentrifuge tube (Table 2).

**Table 2:** Reactions preparation for *in vitro* phage-lipid assay

Negative controls	1x PBS solution	1 mL of PBS solution
	Linoleic acid (0.0009 g/mL)	100 μL of linoleic acid + 900 μL of PBS solution
	Linoleic acid (0.009 g/mL)	10 μL of linoleic acid + 990 μL of PBS solution
	Linoleic acid (0.09 g/mL)	1 μL of linoleic acid + 999 μL of PBS solution
Positive controls	Lambda (5 × 10^10^ PFU/mL)	100 μL of Lambda + 900 μL of PBS solution
	T4 (4 × 10^8^ PFU/mL)	100 μL of T4 + 900 μL of PBS solution
Testing samples	Lambda + Linoleic acid (0.0009 g/mL)	100 μL of Lambda + 100 μL of linoleic acid + 800 μL of PBS solution
	Lambda + Linoleic acid (0.009 g/mL)	100 μL of Lambda + 10 μL of linoleic acid + 890 μL of PBS solution
	Lambda + Linoleic acid (0.09 g/mL)	100 μL of Lambda + 1 μL of linoleic acid + 899 μL of PBS solution
	T4 + Linoleic acid (0.0009 g/mL)	100 μL of T4 + 100 μL of linoleic acid + 800 μL of PBS solution
	T4 + Linoleic acid (0.009 g/mL)	100 μL of T4 + 10 μL of linoleic acid + 890 μL of PBS solution
	T4 + Linoleic acid (0.09 g/mL)	100 μL of T4 + 1 μL of linoleic acid + 899 μL of PBS solution
  b. Incubate at 37°C and shake at 200 rpm for about 1 h.
  c. Centrifuge at 5,000 x g for 10 min at 4°C to fractionize linoleic acid.
  d. Avoid the top layer of linoleic acid, pipette 200 μL of suspension into a 1.5 mL-microcentrifuge tubes.

***Note:*** The suspension contains no phages (for negative controls) or phages that are not bound to linoleic acid.

32. Make a 10-fold serial dilution of suspensions obtained from step 31d.
  a. Pipette 450 μL of LB broth and 50 μL of suspension.
  b. Add 100 μL of exponential growth of *E. coli* and briefly vortex to mix.
  c. Short spin and leave the tubes undisturbed for 10 min to allow phage-host attachment.
33. Perform plaque assay.
  a. Melt top agar and aliquot 5 mL into a 15 mL-tube.
    **CRITICAL:** Keep the tubes at 55°C to prevent top agar from solidifying.
  b. Pipette suspension obtained from step 32c into melted top agar, roll the tubes in hands to mix and prevent bubbles.
  c. Quickly but gently pour the contents onto a petri dish containing solidified bottom agar.
  d. Swirl and leave plates undisturbed for 30 min to set the agar.
  e. Incubate plates at 37°C overnight.
34. Quantify infective phages.
  a. Examine and count the number of plaque-forming units (PFU) on the incubated plates.
  b. Calculate phage titer using the formula below.

$$\text{Titer}\;\left(\text{PFU}/\text{mL}\right)=\left(\text{number}\;\text{of}\;\text{PFU}/\text{volume}\;\text{used}\;\text{in}\;\mu L\right)\;\times \;\left(10^{3}\;\mu L/\text{mL}\right)\;\times \;\text{dilution}\;\text{factor}$$

## Expected outcomes

**Figure 2:**
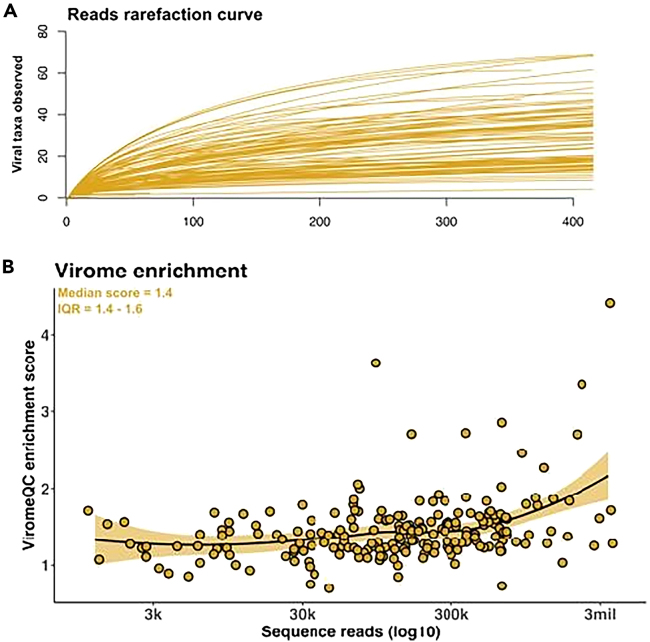
Examination of the phageome sampling completeness produced by our optimized phage isolation and DNA extraction protocol (A) The study samples show flattened rarefaction curves of sequenced reads, indicating a further sequencing would not increases new viral taxa. (B) The length distribution and enrichment scores of assembled viral contigs calculated by ViromeQC, showing an abundant community of phages in the study samples.

The optimized phage isolation and DNA extraction protocol are expected to produce sufficient sampling depth to characterize phageome in low volume preterm HM (Figure 2A). Samples are expected with a median yield of 5.62 × 10^6^ sequence reads and with a viral enrichment score of 1.4 (Figure 2B).

## Limitations

The optimized phage isolation and DNA extraction protocol omits a reverse transcription and multiple displacement amplification step prior to metavirome library preparation. As such, RNA or ssDNA viruses may be under-represented in the data. In addition, the characterization of phages using viral contigs with high nucleotide similarity to viruses in the NCBI viral database, may fail to annotate previously uncharacterized viral entities. Unclassified virus annotation and host prediction using predicted protein approaches may improve phage recovery in future studies of preterm HM.

## Troubleshooting

### Problem 1

Difficulty in extracting phages from the skim milk and milk fat fractions of low volume and low diversity preterm HM.

### Potential solution

Homogenize HM samples in 1x PBS with a minimum ratio of 1:3, followed by an intense horizontal agitation can aid a better separation of skim milk and milk fat, as well as microbes that bound on the membrane of milk fat globules.

### Problem 2

The presence of maternal chromosomal and microbial DNA during phage extraction.

### Potential solution

Add DNase to the isolated phage suspension with a minimum concentration of 12 U/mL and then incubate at 37°C for a minimum of 1 h can deplete most extracellular DNA. We observe no amplification of 16S gene fragments on agarose gel electrophoresis.

### Problem 3

Shotgun metagenomics data processing for phageome study is computational intensive.

### Potential solution

Reduce the computational time and memory by subsetting raw ‘qc_reads’ files into batches of maximum 10 samples within subdirectories and running parallel slurm scripts (step 24c). We also use a custom approach including deduplication (step 25d), filtering assemblies on coverage (step 25f), identification of viral assemblies with VirSorter2 (step 25h) and quality checking with CheckV (step 25i) to replace MetaQUAST.

## Resource availability

### Lead contact

Further information and requests for resources and reagents should be directed to and will be fulfilled by the lead contact, Darren L. Smith (darren.smith@northumbria.ac.uk).

### Technical contact

Technical questions on executing this protocol should be directed to and will be answered by the technical contact, Darren L. Smith (darren.smith@northumbria.ac.uk).

### Materials availability

This study did not generate new unique reagents.

### Data and code availability

The raw metagenomics data generated during this study are available at ENA (PRJEB58774): https://www.ebi.ac.uk/ena/browser/view/PRJEB58774. This publication did not generate any original code. Any additional information required to reanalyze the data reported in this paper is available from the lead contact upon request.

## Acknowledgments

This project is funded by Action Medical Research (AMR) for children (grant reference no. GN2730). We also thank the research nurses, clinical staff, and all participant families at the Royal Victoria Infirmary, Newcastle upon Tyne, UK, for their willingness to allow the use of residual samples. The graphical abstract and figures were created using Biorender.com.

## Author contributions

W.C.Y. detailed the steps in phage isolation and DNA extraction, as well as the *in vitro* phage-lipid assay. W.C. described the protocol for single-phase lipidomic extraction for LC/MS-based discovery lipidomics. G.R.Y. produced scripts of bioinformatics and statistical analysis. All authors reviewed and approved the manuscript before submission.

## Declaration of interests

The authors declare no competing interests.
